# The consequences of hypoglycaemia

**DOI:** 10.1007/s00125-020-05366-3

**Published:** 2021-02-07

**Authors:** Stephanie A. Amiel

**Affiliations:** Department of Diabetes, School of Life Course Sciences, Faculty of Life Sciences and Medicine, King’s College London, London, UK

**Keywords:** Diabetes mellitus, Hypoglycaemia, Review

## Abstract

Hypoglycaemia (blood glucose concentration below the normal range) has been recognised as a complication of insulin treatment from the very first days of the discovery of insulin, and remains a major concern for people with diabetes, their families and healthcare professionals today. Acute hypoglycaemia stimulates a stress response that acts to restore circulating glucose, but plasma glucose concentrations can still fall too low to sustain normal brain function and cardiac rhythm. There are long-term consequences of recurrent hypoglycaemia, which are still not fully understood. This paper reviews our current understanding of the acute and cumulative consequences of hypoglycaemia in insulin-treated diabetes.

**Supplementary Information:**

The online version contains a slide of the figure for download available at 10.1007/s00125-020-05366-3.

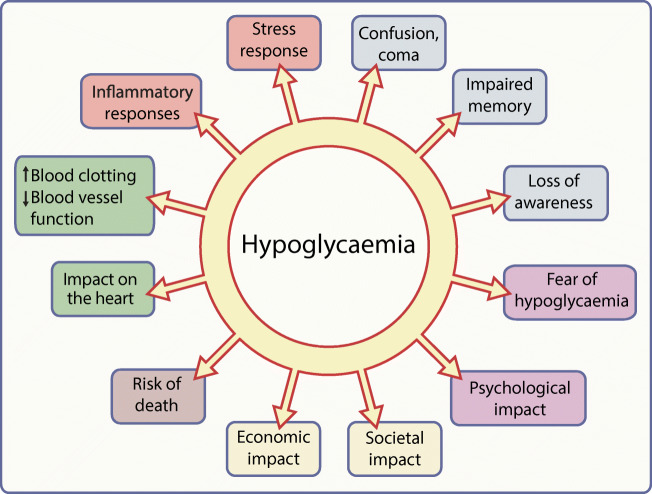

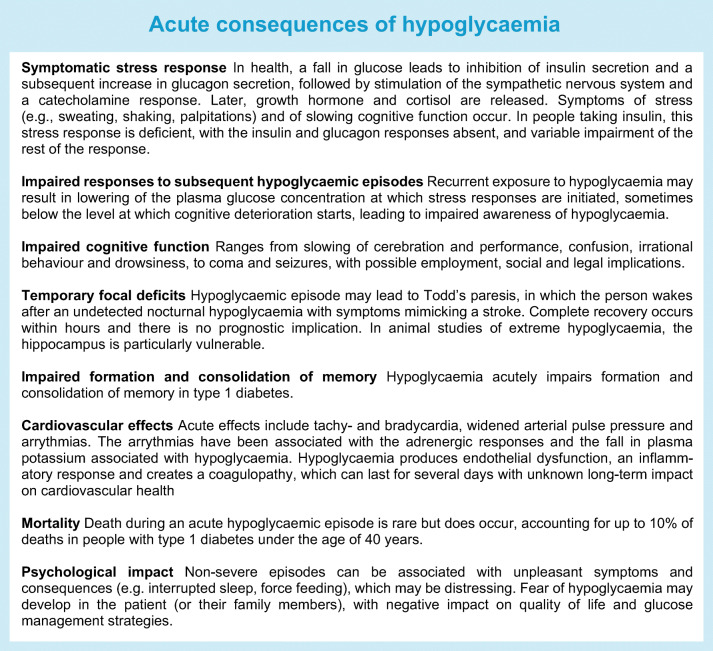

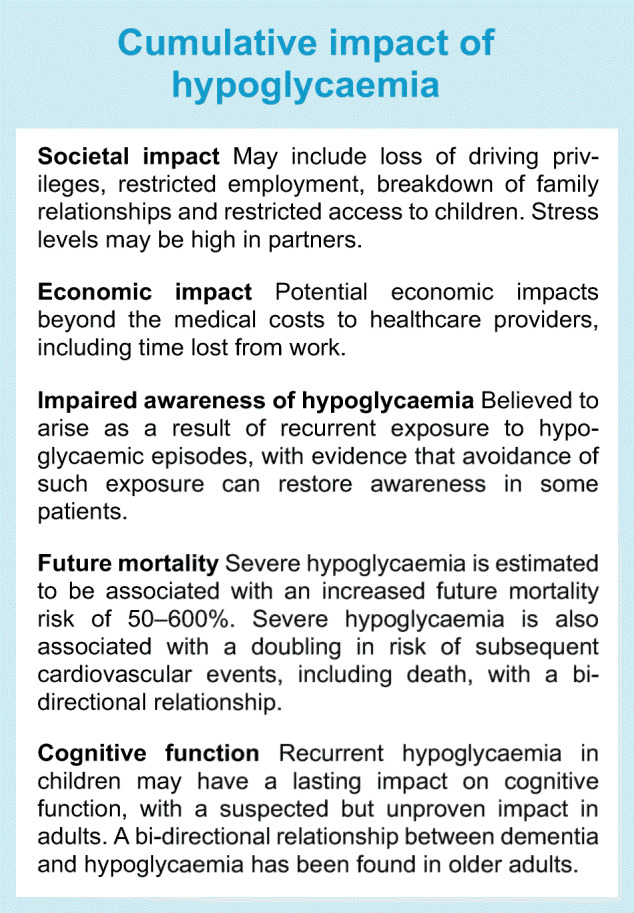





## Introduction

Hypoglycaemia as an adverse consequence of diabetes therapies has been known since the early days of insulin’s discovery. In their book, ‘Breakthrough: Elizabeth Hughes, the discovery of insulin, and the making of a medical miracle’, Thea Cooper and Arthur Ainsberg describe how Elizabeth Hughes, diagnosed with type 1 diabetes in 1919, and her nurse, Blanche ‘shared a bed in the hope that Blanche could better detect and response to a night time attack’ and that Blanche saved Elizabeth’s life ‘dozens of times … with orange juice or a molasses kiss’ [1]. Describing events in 1922, the authors note that ‘several diabetics had suffered hypoglycaemic shock’ [1]. In purifying insulin, James Collip defined a unit of it as the amount that would send a rabbit into hypoglycaemic seizure. One hundred years later, hypoglycaemia remains a risk for everyone taking insulin [1].

## Definitions of hypoglycaemia

Hypoglycaemia is defined as a concentration of glucose in the blood that is lower than normal, traditionally defined biochemically as a plasma glucose of <3.5 mmol/l. A precise biochemical definition is complex, however. Blood glucose concentration is a continuum and the value that is associated with harm depends on the harm that is being described. Homeostatic responses begin as soon as plasma glucose starts to fall, with cessation of endogenous insulin secretion and stimulation of pancreatic glucagon noted at plasma glucose values of around 4 mmol/l. Evidence for material harm, such as impaired cognition, cardiac arrythmia, defective responses to subsequent low glucose values and associations with mortality rate has accrued for a plasma glucose value of less than 3 mmol/l [2]. Such evidence has underpinned a consensus statement from the International Hypoglycaemia Study Group, endorsed by the American Diabetes Association, the European Association for the Study of Diabetes and some charitable and patient groups, which defines three levels of plasma glucose that are important to use when describing low-glucose episodes caused by diabetes therapies (Table 1) [2, 3].

**Table 1:** Levels of hypoglycaemia that should be reported in clinical trials, and which have clinical implications for people with diabetes

Level	Name	Plasma glucose	Implications
1	Hypoglycaemia alert	<3.9 mmol/l^a^	Lower limit of ‘glucose in range’ Usually asymptomatic Treat to prevent hypoglycaemia Consider regimen change if recurrent
2	Clinically important	<3 mmol/l	Associated with impaired cognitive function Repeated episodes cause reduced awareness Predicts severe hypoglycaemia Associated with cardiac arrhythmias Predicts mortality
3	Severe	Not specified	Cognitive decline results in the need for treatment by another person May be further divided to specify episodes requiring parenteral therapy and/or episodes associated with loss of consciousness or seizure

Data from The International Hypoglycaemia Study Group [2]

^a^In the original document, the International Hypoglycemia Study Group (IHSG) had defined level 1 as glucose ≤3.9 mmol/l [2] but this was refined by a subsequent consensus to make a clear distinction between this classification and the lower limit of the desirable glucose range, which is 3.9 mmol/l [3]

For people with diabetes, hypoglycaemia may be better defined by the clinical picture and by the degree of distress and disruption an episode may cause, ranging from having to ingest carbohydrate when not wishing for it, to unpleasant but protective symptoms of an acute stress response, to confusion and coma. The American Diabetes Association categorises hypoglycaemic episodes into: severe (requiring third party intervention because of cognitive impairment too great to support self-treatment); documented symptomatic (also now referred to as non-severe and described as symptomatic episodes confirmed by a low blood glucose measurement and by definition, self-treated); asymptomatic (detected by testing only); probable symptomatic (symptoms of hypoglycaemia but not confirmed by a measurement); and pseudohypoglycaemia (symptoms of hypoglycaemia accompanied by a non-hypoglycaemic blood glucose concentration) [4].

## The acute consequences of hypoglycaemia

### Symptomatic stress response and impaired response to subsequent hypoglycaemic episodes

Hypoglycaemia elicits a stress response, which acts to correct the glucose fall. In health, cessation of insulin secretion and stimulation of pancreatic glucagon, driven by both local and central neuroendocrine signalling, abort a plasma glucose fall through stimulation of hepatic endogenous glucose production. In insulin-deficient diabetes, where exogenous insulin or an insulin secretagogue is being taken, circulating insulin levels remain elevated and pancreatic alpha cells, unable to detect a signal from falling beta cell stimulation, do not secrete glucagon [5, 6]. Correction of the falling glucose is, therefore, dependent on hyperglycaemic sympathetic nerve stimulation, catecholamine secretion and, critically, the person recognising the hypoglycaemia and ingesting carbohydrate. The plasma glucose concentration at which these responses are triggered is influenced by prior glycaemic experience: people with poorly controlled type 2 diabetes and no previous experience of hypoglycaemia may experience some elements of the stress response, and certainly symptoms, at higher plasma glucose concentration than occurs in health [7], and people with previous experience of hypoglycaemia may downregulate the glucose concentration at which sympathetic and hormonal responses to a falling glucose occur, sometimes to a value below that at which cognitive deterioration starts [8, 9]. This creates a syndrome of impaired awareness of hypoglycaemia, in which failure of subjective awareness of hypoglycaemia increases risk of severe hypoglycaemia sixfold in people with type 1 diabetes (in whom severe hypoglycaemia is more common) and 17-fold in individuals with type 2 diabetes who are taking insulin [10, 11]. Parenthetically, we should note that other factors can affect the hierarchy of responses to hypoglycaemia. For example, age can have an impact on response to hypoglycaemia, with findings showing that older people have a lower glucose concentration for subjective awareness and hormonal responses, while the deterioration of cognitive function is preserved in these individuals at an arterialised plasma glucose of 3 mmol/l [12]. Furthermore, in children and in elderly people with diabetes, cognitive and behavioural signs and symptoms may be more prominent in the clinical presentation [13, 14].

We have thus described the first two important acute consequences of hypoglycaemia: the symptomatic stress response and the impairment of responses to subsequent episodes. In addition, we have implied a third: impairment of cognitive function during an acute episode.

### Neurological consequences

The impairment of cognitive function associated with hypoglycaemia ranges from slowing of cerebration and performance, confusion, irrational behaviour and drowsiness, to coma and seizures. Other outcomes include inconvenience, embarrassment, and physical injury to the patient or to others, with possible employment, social and legal implications. There is evidence that the threshold for cognitive impairment changes less in response to antecedent hypoglycaemia than does the threshold for subjective awareness and neurohumoral responses, which provides a potential mechanism for the increased risk of severe hypoglycaemia, as the person becomes unable to self-treat [15, 16].

Other neurological consequences of a hypoglycaemic episode include temporary focal deficits, including Todd’s paresis, in which the person wakes after an undetected nocturnal hypoglycaemia with symptoms and signs mimicking a stroke [17]. Complete recovery occurs within hours and there is no prognostic implication to these episodes. We will discuss below whether residual cognitive deficit results from hypoglycaemia from which an apparently complete recovery is made at the time. Here we note only that in animal studies of extreme hypoglycaemia, the hippocampus seems particularly vulnerable. Anecdotally, memory deficits are reported by people with type 1 diabetes and problematic hypoglycaemia, with some evidence to support an association [18], but hypoglycaemia impairs formation and consolidation of memory in type 1 diabetes acutely [19, 20] and whether the memory deficits are resolved by hypoglycaemia avoidance remains to be determined.

Hypoglycaemia is associated with changes in regional brain activation, not just in the region of the hypothalamic–pituitary axis, but also in brain regions involved in interoception (relevant to symptom generation and perception) and in regions involved in emotional salience, aversion, executive function and memory [21]. Long duration type 1 diabetes can alter these regional brain responses and it has been postulated that enhanced thalamic activity in hypoglycaemia in type 1 diabetes may support subjective awareness in the face of reduced catecholamine responses [22].

### Cardiovascular consequences

Other potential acute consequences of hypoglycaemic episodes include cardiovascular effects, both as a result of the stress response (tachycardia, widened arterial pulse pressure) and arrythmias [23, 24], including lengthening of the QT interval (QTc) on an electrocardiogram and bradycardia. Such arrythmias are associated with the adrenergic responses and the fall in plasma potassium associated with hypoglycaemia [25]. Hypoglycaemia produces endothelial dysfunction (as does hyperglycaemia) [26], an inflammatory response [27], and creates a coagulopathy, which can last for several days [28].

### Mortality

Death during an acute hypoglycaemic episode is rare but does occur, accounting for up to 10% of deaths in people with type 1 diabetes under the age of 40 years, in whom other causes of death are, happily, rare [29].

## Nocturnal hypoglycaemia

Over 50% of severe hypoglycaemic episodes in insulin-treated diabetes occur at night and this topic has been reviewed previously [30]. Hypoglycaemia at night has been shown to impact on recognition of hypoglycaemia the next day [31] and, as already discussed, may have an impact on the consolidation of memory that should happen during sleep [20]. The counterregulatory responses to hypoglycaemia are suppressed during deep sleep, so episodes may remain asymptomatic and undetected [32]. There is little evidence for an impact on cognitive function the next day, but mood and well-being have been described as adversely affected [30]. Rare, unexpected nocturnal deaths have been reported in young people with type 1 diabetes, attributed to hypoglycaemia and accounting for 5% of all deaths in this population [33]. The physiological and psychological impact of asymptomatic nocturnal hypoglycaemia that is recognised on waking by people using retrospective intermittently monitored glucose meters vs hypoglycaemia detected at the time by alarms from real-time continuous glucose monitoring remains to be determined.

## The evolution of acute responses to hypoglycaemia over time

The main driver of hypoglycaemia in diabetes is glucose lowering therapy: exogenous insulin or insulin secretagogues, such as sulfonylureas. Their effects are exacerbated by the defects in the counterregulatory responses to a falling blood glucose that occur in insulin-deficient diabetes (described above), including the loss of glucagon responses and, later, impaired sympathetic and catecholamine responses [5]. Severe hypoglycaemia occurs at a rate of 1.3 episodes per person per year in people with type 1 diabetes; but its occurrence is extremely skewed, with only 40% of individuals experiencing an episode in any one year, and 10% of adults with type 1 diabetes contributing nearly 70% of all episodes in the year, with many of these people reporting recurrent events [34]. Interestingly, a recent study of people with insulin-treated type 2 diabetes in the Netherlands described only slightly lower prevalence of severe hypoglycaemia (32%) [35]. Impaired awareness of hypoglycaemia is a major risk factor in both types of diabetes.

## The cumulative impact of hypoglycaemia

The common outcome of any hypoglycaemic episode is complete recovery, but we have already begun to allude to possible impacts of hypoglycaemia on future health.

### Psychological, societal and economic impact

There is a psychological impact from hypoglycaemia experiences. Any episode can be unpleasant: non-severe episodes can be associated with unpleasant symptoms, which may be distressing to the person experiencing them to the extent that the individual will describe the episode as ‘very severe’. One person with diabetes described multiple negative experiences of hypoglycaemia including interruption of activities, sleep disruption and a ‘horrible force feeding one has to endure if not remotely hungry’ (personal communication with author). Fear of hypoglycaemia may develop and lead to behaviours that are unhelpful either to diabetes control or to normal social interaction [36]. One recent study described 40–50% of a clinic population of adults with type 1 diabetes as expressing fear of hypoglycaemia [37] and, in people with type 2 diabetes, non-severe hypoglycaemia may have as big an impact on fear of hypoglycaemia as severe episodes, with negative impact on quality of life and on glucose management strategies [38, 39]. Other family members, perhaps particularly parents of children with diabetes, are also affected by fear of hypoglycaemia [40], with partners of people with problematic hypoglycaemia (impaired awareness of hypoglycaemia and recurrent severe episodes) expressing very high distress [41]. The societal impact may include loss of driving privileges, restricted employment, and breakdown of family relationships, with restricted access to children. There are potential economic impacts beyond the costs to healthcare providers (who are involved in a tiny minority of cases), including time lost from work, that have still to be fully understood [42].

### Impaired awareness of hypoglycaemia

Impaired awareness of hypoglycaemia is believed to arise as a result of recurrent exposure to hypoglycaemic episodes (under 3 mmol/l), with evidence that avoidance of such exposure can restore awareness, both in mechanistic studies (e.g. [43]), and clinically [44]. McCrimmon’s group has described this as a conditioning response [45]. A small but highly vulnerable group of patients seem resistant to interventions for hypoglycaemia avoidance [46]. Some may have unhelpful cognitions around hypoglycaemia, which may act as barriers to future hypoglycaemia avoidance [47, 48]. One clinic-based study of people with type 1 diabetes found that one-third of people at high risk for severe hypoglycaemia expressed low concern about their hypoglycaemia [49]. Neuroimaging studies offer a potential explanation, with altered activation of brain regions involved in appetite control, emotional salience, aversion, recall, arousal and decision making in response to hypoglycaemia in people with established impaired awareness and recurrent severe hypoglycaemia [50, 51]. Interventions that target cognitions around hypoglycaemia may be helpful in this group [52].

### Risk of future mortality

There is a growing body of evidence linking severe hypoglycaemia to future mortality both in hospital and in the community, with estimates of increased risk ranging from 50% to 600% [53]. Some of the responses to acute hypoglycaemia, particularly the proinflammatory and coagulopathy effects, have been implicated as underlying mechanisms, but the data remain compatible with hypoglycaemia being a marker of frailty and high risk of death [53]. Severe hypoglycaemia has been associated with approximately a doubling in risk of subsequent cardiovascular events, including death, but the relationship is bi-directional [54].

### Cognitive function

Meanwhile, concern remains that recurrent hypoglycaemia may have a lasting impact on cognitive function. There is some evidence that recurrent severe hypoglycaemia in children at an age when the brain is still developing may result in subtle impairment of performance on cognitive function testing in adolescence, especially when seizures have accompanied the hypoglycaemia [55, 56]. In adults data remain controversial, with consistently reassuring data coming from long-term follow-up of the DCCT/Epidemiology of Diabetes Interventions and Complications (EDIC) cohort of type 1 diabetes patients and from the Outcome Reduction With Initial Glargine Intervention (ORIGIN) trial in people with type 2 diabetes [57, 58]. However, a recent large study in older adults with type 1 diabetes demonstrated an association between patient-reported history of severe hypoglycaemia events (episodes resulting in emergency department or inpatient admission), either recent (any) or life-time exposure, and impaired performance in global cognition, language and executive function and recent episodic memory, with evidence for a dose effect [59]. An earlier meta-analysis also found a bi-directional relationship between dementia and hypoglycaemia in older adults [60].

## Conclusions

**Fig. 1:**
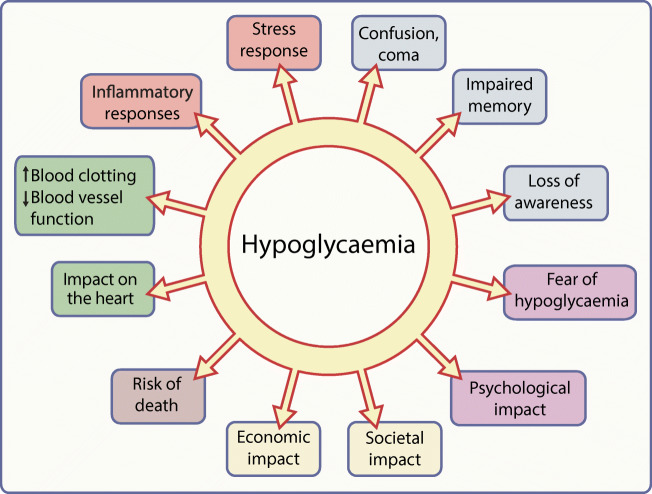
A graphical summary of the potential consequences of hypoglycaemia. Elements of the stress response to a hypoglycaemic episode are shown in orange text boxes; other colours indicate different classes of possible consequences of hypoglycaemic episodes and of hypoglycaemia itself (blue, neurological/cognitive; purple, psychological; yellow, socioeconomic; brown, mortality; green, cardiovascular). This figure is available as a downloadable slide

Hypoglycaemia has been a complication of diabetes treatments since the discovery of insulin, and remains a major concern for people with diabetes, their families and friends, and healthcare professionals and providers. Experiencing hypoglycaemia has consequences, as summarised in Fig. 1. Individual episodes of hypoglycaemia are inconvenient, unpleasant and potentially dangerous and recurrent episodes have long-term negative health implications that are still being explored [61]. Minimising hypoglycaemic exposure must remain a key target for diabetes therapies and hypoglycaemia experience must always be a metric for assessing the effectiveness of diabetes management.

## Supplementary information

Figure slide (PPTX 209 kb)
