# Supplementary material for: The consequences of hypoglycaemia
Source: Diabetologia. 2021 Feb 7;64(5):963–70. doi: 10.1007/s00125-020-05366-3 (PMC8012317; doi:10.1007/s00125-020-05366-3)
Supplement: Supplementary file 1 — (PPTX 209 kb) [file 125_2020_5366_MOESM1_ESM.pptx]

## Slide 1
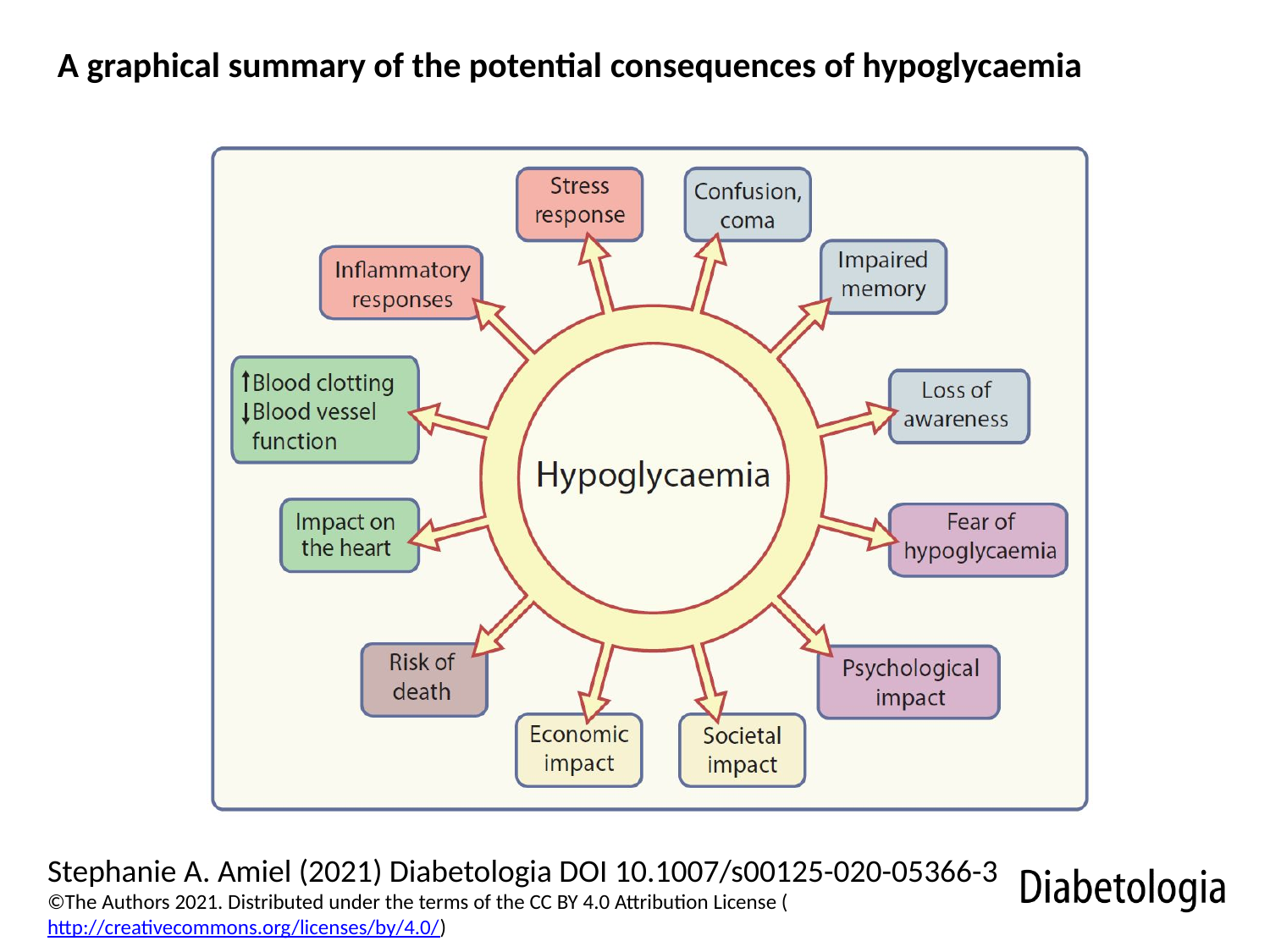

A graphical summary of the potential consequences of hypoglycaemia
Stephanie A. Amiel (2021) Diabetologia DOI 10.1007/s00125-020-05366-3
©The Authors 2021. Distributed under the terms of the CC BY 4.0 Attribution License (http://creativecommons.org/licenses/by/4.0/)
